# Exploring Consumers’ Acceptance, Emotions, and Attitudes Toward Clean Label Chocolates and/or Added Cocoa Shell

**DOI:** 10.1111/1750-3841.71360

**Published:** 2026-08-17

**Authors:** Fernanda Schiehll Azevedo, Voltaire Sant'Anna, Juliana Santos, Ludmylla Tamara Crepalde, Marc François Richter

**Affiliations:** ^1^ Life and Environmental Area State University of Rio Grande do Sul, Encantado campus Encantado Brazil; ^2^ Department of Food Technology Federal University of Viçosa Viçosa Brazil

## Abstract

The influence of cocoa shells and/or the absence of additives in chocolates has not been deeply explored regarding consumers’ behaviors, and the present work provides essential information to help the food industry design strategies to produce healthy and sustainable products. The objective of the present work was to evaluate the impact of the addition of cocoa shell to chocolate and the removal of additives from its formulation on consumer behavior. Participants (*n* = 135) evaluated chocolate samples (base [B], added cocoa shell [CS], without additives [CL], and cocoa shell without additives [CS + CL]) regarding acceptance, evoked emotions, and perceived sensory attributes in blind and stimulated conditions. In addition, the present work explored the preferences and drivers of liking of samples using the hard‐laddering technique. CL affected acceptance, while CS did not influence liking scores, and CL evoked more positive emotions than CS in blind tests. In stimulated tests, when CL elements were presented on front‐of‐package labeling, the presence of an indication of CS positively impacted the expected liking; meanwhile, when CL elements were not presented on label, the indication of CS did not influence expected liking. In addition, the presence of CS on labels evoked more positive emotions than when it was not present, and CL slightly impacted emotional responses in the stimulated conditions. The most preferred chocolate was the combination of CL and CS, and the main purchasing driver was because there was more information about the product on front‐of‐package labeling.

**Practical Applications:**

SabsThe present work brings important information to design marketing and novel product strategies regarding upcycled foods and the addition of chemicals to improve food quality by deepening exploration of consumers’ senso‐affective responses (acceptance, emotions, perceived attributes, and drivers of purchasing) toward chocolates added of cocoa by‐products and clean label bars. Results are important not only to chocolate factories but also to the whole food industry chain.

## 1 Introduction

Chocolate is one of the most appreciated groceries worldwide, with a market size of $140.12 billion by 2025, and is expected to grow annually by 4.89% up to 2030 (Singh 2022). Consumers are willing to pay premium prices for chocolates with superior taste, better ingredients, and unique flavors, but consumers have become healthier and more sustainable, which has challenged industries to change chocolate formulations to offer healthier and more sustainable options in the market (Singh 2022; Carvalho Machado Kamphorst et al. 2025).

The circular economy within food industries has become more than a market trend, but much needed to combat the impacts of the food production chain on climate change. Upcycling foods may be defined as the utilization of food by‐products, after proper treatment, in food formulations for human consumption (Aschemann‐Witzel et al. 2023; Carlini et al. 2023). Valorization of food by‐products based on this strategy has been the focus of several scientific studies, since they are usually destined for less noble utilization such as animal feeding, composing, or burning for fuel in food industries, but present unexplored nutritional value (Grillo et al. 2019; Carlini et al. 2023; Punia Bangar et al. 2024). In the chocolate production chain, cocoa shells (also called hulls or husks) represent approximately 10%–17% of the total cocoa bean weight, (Grillo et al. 2019; Rojo‐Poveda et al. 2020). This residue is rich in polyphenolic compounds with antioxidant activities and fibers and is a potential source of functional food and beverage ingredients (Grillo et al. 2019; Rojo‐Poveda et al. 2020). Owing to their health benefits, cocoa shells have been proposed to be added to food formulations, helping to enhance the fiber and polyphenolic content in cookies, implying greater sensory acceptance of the bakery product (De Barros et al. 2020). Rinaldi et al. (2020) observed cocoa shells to be an interesting ingredient in gluten‐free breads, leading to a pleasant darker color with slight changes in texture. When this by‐product was added to the chocolate bar formulation, the results indicated that it slightly impacted the polyphenolic content and produced softer and darker samples, which may be a positive aspect for consumers (Barišić et al. 2020). However, the chocolate particle size distribution and rheological features were negatively affected, indicating that further research is essential to minimize these problems (Barišić et al. 2020).

In a healthy context, consumers have highly valued foods without chemical additives (preservatives and stabilizers) due to toxicological concerns about their ingestion (Rivaroli et al. 2020; Cao and Miao 2023). Clean label foods have emerged because they are produced with a reduced number of ingredients, mainly synthetic ones, and are thus perceived as more natural, less processed, and more nutritious (Almeida Sá et al. 2023; Cao and Miao 2023). Consumers perceive foods with fewer ingredients disposed in front‐of‐package label (FOPL), familiar ingredients, and without additives as healthier, more socially responsible, and with superior sensory appeal (Cao and Miao 2023). Chen et al. (2022) claimed that clean labels work as a mental shortcut (heuristic) for consumers, who associate shortlists and simple ingredients with safer, less processed, and healthier choices, even if this does not always reflect the nutritional or food safety reality, being an important marketing strategy for food industries to leverage food sales. However, the removal of chemical additives from food formulations is a major challenge for the food industry in terms of sensory and shelf‐life aspects. Lecithin and polyglycerol polyricinoleate (PGPR) are emulsifiers used in chocolate formulations to stabilize and homogenize the dough, affecting moisture, temperature sensitivity, and tempering behavior, as well as bloom stability, fat migration, and oxidation of chocolate in solid form, among other benefits (Schantz and Rohm 2005; Sözeri Atik et al. 2020). In these scenarios, the proposal of a clean label chocolate is scarce in the literature, and further work is essential for its proper adoption by the food industry. In addition, exploring cocoa shells in chocolates needs to go beyond liking aspects, since consumers’ food choices are complex, and non‐sensory cues play critical roles in perception, attitudes, and emotional responses, affecting acceptance and preferences (ARES et al. 2010; Henn et al. 2023; Carvalho Machado Kamphorst et al. 2025).

In this context, FOPL plays a critical role (Asioli et al. 2017; Cao and Miao 2023), since it affects consumers’ expectations. According to expectancy theory (Anderson 1973), consumers integrate prior information with sensory input during consumption, leading to assimilation or contrast effects depending on the congruence between expectations and actual product experience (Ares et al. 2010). Studies have demonstrated that FOP labels such as “reduced sugar,” “organic,” or warning labels can significantly alter liking scores, perceived sweetness, bitterness, and purchase intention, even when products are identical (Asioli et al. 2017). Furthermore, food labels can evoke different perceptions and emotional responses, which are strongly associated with food choice and consumer acceptance (Gutjar et al. 2015; de Alcantara et al. 2025). These effects are particularly relevant for chocolate because consumption is closely linked to multisensory pleasure, indulgence, and emotional gratification, making consumers highly susceptible to contextual and informational influences conveyed by packaging. However, these features have been little explored within chocolates, although they one of the most appreciated groceries worldwide.

Thus, the objective of the present work was to evaluate the impact of the addition of cocoa shell to milk chocolate and the removal of additives from its formulation on acceptance, emotions evoked, and perceived sensory attributes. In addition, the present work explored preferences, emotional responses, and drivers of liking FOPL, which claimed clean labeling and/or addition of cocoa shell.

## 2 Materials and Methods

### 2.1 Materials

Sugar (Raízen, Rio de Janeiro, Brazil), milk powder (CCGL, Cruz Alta, Brazil), cocoa butter (Barry Callebaut, Zurich, Switzerland), cocoa liquor (Cargill, Minnesota, USA), cocoa shell powder (Tovani Benzaquen São Paulo, Brazil), soy lecithin (IFF, Esteio, Brazil), PGPR (Concepta, Valinhos, São Paulo, Brazil), and liquid vanillin (IFF, Esteio, Brazil) were donated by a local chocolate‐manufacturing company.

According to the manufacturer's data sheet, cocoa shells were prepared from roasted cocoa shells after cleaning and maceration (mechanical grinding to 100 µm). Thermal processing consisted of reaching 100°C–120°C for 8 min and maintaining a brown color and characteristic flavor. Each 100 g of cocoa almond shell contained 53 g of dietary fiber, 19 g of protein, and 4.5 g of fat.

### 2.2 Chocolate Production

Chocolate trials were produced following a full factorial design using cocoa shell and clean labels as independent variables, as shown in Table 1. Milk chocolate was chosen as model, since it is the most appreciated among Brazilian consumers (Nexus 2025). Detailed information on the chocolate formulations is provided in Table S1. Thus, four different chocolate bars were tested: (i) base samples (B) were produced by the combination of sugar, cocoa butter, cocoa liquor, powdered milk, lecithin, PGPR, and vanillin; (ii) in cocoa shell samples (CS), powdered cocoa shells were added to 4% of the formulation and the cocoa liquor amount was reduced based on previous tests to obtain a homogeneous chocolate bar; (iii) in the clean label sample (CL), lecithin, PGR, and vanillin were removed from the control formulation; and (iv) in CS + CL samples, powdered cocoa shells were added to the CL formulation.

**TABLE 1:** Factorial design with code variables (real variables) to produce chocolate samples for consumers’ tests.

Sample	CS	CL
B	−1 (absence—without CS)	−1 (No—with additives)
CS	+1 (presence—with CS)	−1 (No—with additives)
CL	−1 (absence—without CS)	+1 (Yes—without additives)
CS + CL	+1 (presence—with CS)	+1 (Yes—without additives)

Abbreviations: B, base sample; CL, clean label; CS, cocoa shell.

Chocolate production followed the recommendation of Cemin et al. (2023), with minimal modifications. Each formulation was produced in 5 kg batches in a Bühler pilot plant. A mixture of sugar, milk, cocoa bean shells, melted liquor, and butter was refined to particle sizes between 20 and 25 µm. At this stage, fat content was maintained at 24%. Conching was performed for 4 h at 60°C with shaking at 80 rpm. After 3 h of conching, the remaining fat, emulsifiers, and flavors were added (Becket 2008). The chocolate mass was stored in a sealed container and in thermal boxes for later processing. Tempering was performed as follows: (i) the chocolate was melted at 45°C to remove the crystal history, (ii) approximately two‐thirds of the chocolate mixture was poured onto a marble slab and mixed with a flexible spatula until the product reached 27°C to produce seed crystals, and (iii) this cooled portion was then mixed with the remaining one‐third of the warm chocolate (40°C) to melt the unstable crystal polymorphs and reach the chocolate working temperature of 31°C–32°C. The tempered chocolate was poured into a 10 g plastic mold with the following dimensions: 34 mm × 23 mm × 14 mm. Subsequently, it was cooled to 10°C for 30 min for solidification. The chocolate bars were unmolded and wrapped in a lead paper. The samples were thermally insulated at 24°C.

### 2.3 Consumers’ Tests

#### 2.3.1 Ethical Aspects

The present work followed the ethical procedures of the Helsinki Declaration and was previously approved by the Ethics Committee of the State University of Rio Grande do Sul under protocol number 7.098.862 and the Certificate of Presentation of Ethical Appreciation number 81953724.1.0000.8091. Before the consumer study, all volunteers read and signed the Free and Informed Consent Form, agreed to participate in the study, and no financial compensation was provided. No information regarding chocolate formulation was provided.

#### 2.3.2 Participants

Participants (*n* = 135) were recruited via social media and met the following criteria: regularly consume milk chocolate; over 18 years old; no intolerance or allergies to milk protein, lactose, or nuts; not pregnant or lactating; and liked to consume milk chocolates. Age and sex quotas were not included.

#### 2.3.3 Data Collection

The consumers’ test section was composed of a blind test, followed by a FOPL stimuli test. In the blind tests, responses toward acceptance and evoked emotions were raised. In the second step, fictitious FOPL was presented to consumers, and expected liking, emotions evoked, willingness to purchase, and laddering tests were performed.

##### 2.3.3.1 Blind Test

The consumer study was conducted as a central location test in a private room in Lajeado (RS, Brazil), where participants attended groups of up to 20 people. Samples (10 g, 34 mm length × 23 mm width × 14 mm height) at room temperature (∼20°C) were served monadically and presented in a random and balanced way onto odor‐free plastic dishes coded with three‐digit random codes, under white lighting in a neutral/central location test room (∼20°C). The participants were instructed to clean their palates between samples with filtered water. Answers were collected using Google Forms software (California, USA), with the sample codes presented at the beginning of each online form section, followed by hedonic, emotional, and sensory attributes tasks (Carvalho Machado Kamphorst et al. 2025). Consumers used their mobile devices for data collection.

The consumer test consists of four steps. Initially, chocolate samples were presented in blind conditions, and consumers were asked to rate their acceptance after tasting them using a 9‐point structured scale (1 = *dislike extremely*, 9 = *like extremely*). Then, emotional responses were obtained using the check‐all‐that‐apply (CATA) methodology by asking consumers to check all emotions that were applied when they tasted each sample from the EsSense 25 profile list (Nestrud et al. 2016) with a few modifications. The emotion term “pleasant” and “joyful” were removed since it presents similar meaning to “happy” (Jaeger et al. 2019); “bored,” “wild,” and “warm” were removed due to error in the translation process from English to Portuguese; “affectionate,” from the EsSense Profile (King and Meiselman 2010) was added because authors consider a relevant emotion to this product category.

##### 2.3.3.2 Front‐of‐Package Labeling Stimuli

In the second step, four fictitious chocolate packages were presented to consumers following the chocolate hypothesis developed in Section 2.1. They were developed using Canva software inspired by Lindt chocolate packages (https://www.chocolate.lindt.com/excellence/new‐excellence‐cocoa‐pure) and are presented in Figure S1. Figure S1A–D represents the base chocolate sample, the chocolate added to CS, the CL chocolate (specifying the three ingredients in the formulation, cocoa butter, and liquor that can be designed as cocoa onto FOPL), and the combination of CS and CL.

Chocolate samples were presented in the same order and conditions of the blind section, and the printed packages (17 cm length × 7 cm wide) were in front of each sample. Consumers were verbally told that the piece of chocolate was from the package in front of it. After tasting each sample, consumers were asked to rate their expected liking using a 9‐point structured scale, anchored in *I would like very much*, and *I would dislike very much*. Then, the emotion list used in Section 2.3.3.1 was presented, and consumers were asked to check emotions evoked when they saw each package.

The hard laddering task was then performed following the procedures of Polizer Rocha et al. (2019). From the packages presented, consumers were asked to choose one chocolate to buy and explain the reasons for this choice using a series of why questions (“why this attribute is important for you?”).

#### 2.3.4 Data Analysis

Analysis of variance (ANOVA) and Tukey, Shapiro–Wilk, Hartley's maximum *F*, Box–Cox transformation, and generalized linear model (GLM) tests were performed using R software version 4.0.5. The hierarchical map was configured using LadderUX software, and the *α* risk was set at 5%.

ANOVA model followed Equation (1).

(1) $$Y_{ijk}=\mu +A_{i}+B_{j}+{\left(AB\right)}_{ij}+\varepsilon_{ijk}$$

where $A_{i}$ is the effect of CL, $\;B_{j}$ is the effect of CS, $\;{\left(AB\right)}_{ij}$ is the interaction between factors (CL and CS), and $\;\varepsilon_{ijk}$ is the random error.

##### 2.3.4.1 Liking Scores

The effect of the presence or absence of cocoa shell or clean label elements and the interaction of CS × CL on chocolates’ acceptance was evaluated by means of liking or expected liking comparison by two‐way ANOVA with randomized complete block design (RCBD), considering each consumer as a block (cocoa shell and clean label variables) followed by Tukey's honest significant difference (HSD) post hoc test. In the ANOVA model, clean label and cocoa shell were considered as fixed effects. The panelists (consumers) were considered random blocks to account for individual variability and to improve the accuracy of the fixed‐effect estimates. Normality of the residuals and homogeneity of variances were evaluated using Shapiro–Wilk and Hartley's maximum *F* tests, respectively.

##### 2.3.4.2 Emotions

Since emotions were evaluated by binary data, a nonparametric approach, the effect of CS, CL, and interaction (CS × CL) on each emotion term evoked in blind and FOPL stimulated tests were evaluated using a GLM, as described in detail by Dupas de Matos et al. (2025). Analysis of deviance and associated chi‐squared tests were used to determine the statistical significance between the main effects and interactions. When significant effects were found, pairwise comparisons were performed using estimated marginal means (emmeans) with *p*‐value adjustment using the Tukey method, with results back‐transformed from the logit to the response (probability) scale.

##### 2.3.4.3 Hard Laddering

The qualitative answers for each individual's laddering task were performed as described by Polizer Rocha et al. (2019). Each consumer sentence was categorized in such a way that terms with similar meanings were grouped in the same dimension and categorized based on consequence and value levels of abstraction based on Barrena et al. (2015).

To verify whether the distribution of choices differed significantly, the Chi‐square goodness‐of‐fit test was performed, and pairwise comparisons were conducted using the Bonferroni correction to identify significant differences between samples. Additionally, adjusted standardized residuals were examined to determine the samples that contributed the most to the observed chi‐square statistics. The hierarchical map was configured using the chosen chocolate, and the cut‐off point for the number of participants was set at 5% (Dall’Ácua et al. 2024).

## 3 Results and Discussion

### 3.1 Consumers’ Profile

The consumer profiles are presented in Table S2. Most of the consumers were women (61.5%, *n* = 83) and had an average age of 38.2 ± 10.1 years. Moreover, most had finished college or presented a higher educational level (72.6%, *n* = 98) and had a monthly income higher than three Brazilian minimum salaries (63.7%, *n* = 86). Regarding the frequency of chocolate consumption, 68.1% (*n* = 92) consumed chocolates at least once a week.

### 3.2 Blind Tests

#### 3.2.1 Acceptance

**FIGURE 1:**
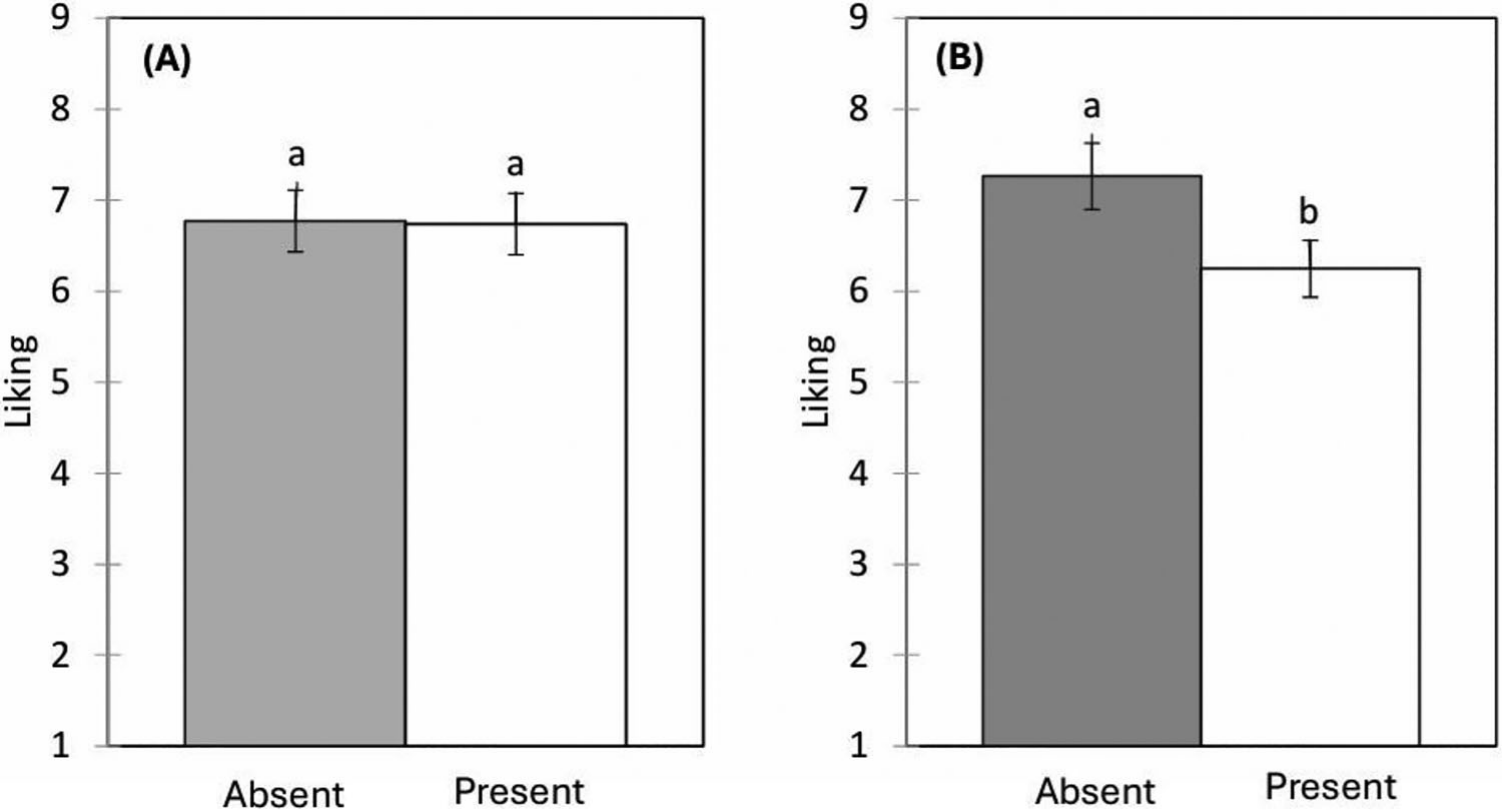
Chocolates’ acceptance in relation to the presense of CS (A) and CL (B). Different letters indicate significant difference between the presence (gray bars) or absence (white bars) of the variable (CS or CL) by Tukey's test.

Consumers’ acceptance of chocolates was neither affected by CS (*p* = 0.814) nor by the interaction of both variables (CS × CL, *p* = 0.159) but was significantly affected by CL (*p* < 0.0001), indicating that the effect of clean labels was consistent regardless of the presence or absence of cocoa shell. Figure 1A shows that the addition of cocoa shell implied on average liking scores of 6.7 ± 0.2, meanwhile the absence of lecithin, PGPR, and vanillin reduced significantly liking scores from 7.3 ± 0.2 to 6.3 ± 0.1 (Figure 1B). This suggests that the addition of cocoa shell, at the levels tested, does not compromise overall liking, supporting its potential as a sustainable ingredient in chocolate formulations. Lecithin and PGPR are important additives in chocolate formulations to improve the texture of the grocery (Peker et al. 2013), as they may decrease viscosity (Nebesny and Żyżelewicz 2005). Chocolates with high viscosity have a pasty mouthfeel that persists in the mouth (Beckett 2008). Viscosity influences flavor “by‐mouth” and taste intensity during consumption (Denker et al. 2006). Schouteten et al. (2018) observed that texture attributes are closely related to overall acceptance, which helps explain the present results. In addition, the absence of vanillin may impact sweet and fruity notes in chocolates, which have been described as drivers of liking for the overall flavor (Cemin et al. 2023), which helps to explain lower acceptance of CL chocolates.

#### 3.2.2 Emotions Evoked

**FIGURE 2:**
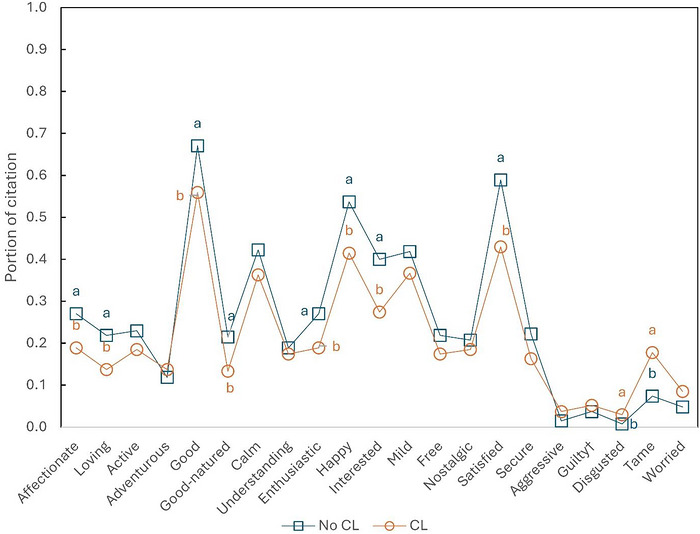
Portion of citation of emotions evoked in blind sensory test between the presence (orange line) and absence (blue line) of CL in the chocolate formulations. Different lower case letters are significantly different across presence/absence of CL at 5% level according to Tukey's HSD test. When letters are not shown, it means not significant. † indicates significant differences between CS elements within that emotion term.

Differences among the different formulations for emotions evoked during the blind test are shown in Table 2, and Figure 2 shows the portion of citation of emotions evoked between the presence and absence of CL.

**TABLE 2:** Deviance and associated *p*‐values from GLM associated with effect of cocoa shell (CS), clean label (CL), and their interaction by emotion for chocolate samples in blind tests. Bold values indicate significance at 5%.

Emotions	CS		CL		CS × CL	
	Deviance	*p*‐value	Deviance	*p*‐value	Deviance	*p*‐value
Affectionate	1.524	0.2171	**5.088**	**0.024**	0.134	0.715
Loving	0.821	0.365	**6.177**	**0.013**	0.744	0.389
Active	0.045	0.832	1.625	0.202	0.038	0.844
Adventurous	0.017	0.897	0.416	0.519	0.407	0.523
Good	0.031	0.859	**7.060**	**0.007**	0.495	0.482
Good‐natured	1.881	0.170	**6.283**	**0.012**	0.537	0.464
Calm	1.123	0.289	1.990	0.158	1.188	0.276
Understanding	0.449	0.503	0.200	0.655	0.764	0.382
Enthusiastic	0.6767	0.411	**5.088**	**0.024**	0.269	0.604
Happy	1.697	0.193	**8.106**	**0.004**	0.886	0.347
Interested	0.304	0.582	**9.623**	**0.001**	0.759	0.384
Mild	0.031	0.860	1.522	0.217	0.000	0.997
Free	0.754	0.385	1.693	0.1932	0.534	0.465
Nostalgic	0.000	0.000	0.423	0.516	0.000	0.000
Satisfied	0.373	0.542	**13.760**	**<0.001**	2.190	0.139
Secure	0.431	0.511	3.058	0.080	0.547	0.458
Aggressive	0.000	1.000	2.725	0.099	1.480	0.224
Disgusted	1.687	0.194	**3.923**	**0.048**	0.467	0.494
Tame	0.622	0.430	**13.542**	**<0.001**	1.351	0.245
Guilty	**6.566**	**0.010**	0.701	0.403	0.251	0.617
Worried	0.479	0.489	3.013	0.083	2.618	0.105

In blind tests, CL and CS × CL did not significantly influence none of the emotion terms, for exception of “Guilty” which showed that the presence of the by‐product evoked higher citation (*p* = 0.010) of this emotion (Table 2). “Guilty” is classified as with no clear classification emotions, and it may present either a positive or negative valence, depending on food type and consumption context (King and Meiselman 2010). The results herein indicate “Guilty” present a neutral connotation, since CS did not impact acceptance (Figure 1A).

Regarding CL, Table 2 showed that “Affectionate” (*p* = 0.024), “Loving” (*p* = 0.013), “Good” (*p* = 0.007), “Good‐natured” (*p* = 0.012), “Enthusiastic” (*p* = 0.024), “Happy” (*p* = 0.004), “Interested” (*p* = 0.001), “Satisfied” (*p*<0.001), emotions with positive connotation (King and Meiselman 2010), were affected positively by the presence of additives in the chocolate formulation (clean label samples), meanwhile “Disgusted” (*p* = 0.048), with negative connotation (King and Meiselman 2010) was effected negatively by the absence of the additives (CL samples). Higher liking scores are usually linked to positive emotions, whereas lower scores are associated with negative connotations (Nestrud et al. 2016; Schouteten et al. 2018), which is in line with the findings of the present study. “Tame” (*p* < 0.001), whose connotation depends on food and context (King and Meiselman 2010) was highly cited when clean label chocolates were evaluated, indicating a negative valence, since clean label samples implied on lower acceptance scores (Figure 1B). “Tame” was observed to be a neutral emotion by Schouteten et al. (2018) when milk chocolates were tested by Belgian consumers, meanwhile for Australian consumers this emotion term was not significant to compose the emotional lexicon to describe chocolates (Gunaratne et al. 2019), indicating an important cultural oriented bias of this emotion. Chocolate texture attributes (creaminess and hardness) were related to overall acceptance and positive emotions such as “Active,” “Pleased,” “Whole,” and “Enthusiastic,” meanwhile chocolate samples with low acceptance evoked negative emotions such as “Worried” and “Disgusted” (Schouteten et al. 2018). Texture is the dominant quality characteristic for several food types (Bourne 2002), and the results of the present study indicate that texture may influence both acceptance and emotions.

Figure 2 also shows a high frequency of citations of positive emotions, while fewer negative emotions are cited. It is well documented that eating is a positive experience, and actual food products are meant to please consumers, implying higher positive emotions than negative ones when foods are consumed (King and Meiselman 2010). Thus, positive emotions (“Happy,” “Satisfied,” “Interested,” and “Enthusiastic”) may also represent valuable marketing tools for the chocolate industry, since emotionally positive food experiences are strongly linked to consumer engagement, purchase intention, brand attachment, and product memorability. “Happy” and “Satisfied” emotions are associated with hedonic gratification and consumption pleasure, which can reinforce brand loyalty and encourage repeated purchases. Meanwhile, “Interested” and “Enthusiastic” indicate curiosity, excitement, and cognitive engagement toward the product, emotions that are particularly relevant for innovative products such as chocolates formulated with cocoa shell or clean label claims. From a marketing perspective, these emotional responses can be strategically explored in product positioning, packaging communication, storytelling, and advertising campaigns to strengthen consumers’ emotional connection with the product. Furthermore, emphasizing emotional benefits alongside sustainability or clean label attributes may help industries increase consumer acceptance and reduce potential resistance commonly associated with reformulated products.

### 3.3 Stimulated Tests

#### 3.3.1 Expected Liking

**FIGURE 3:**
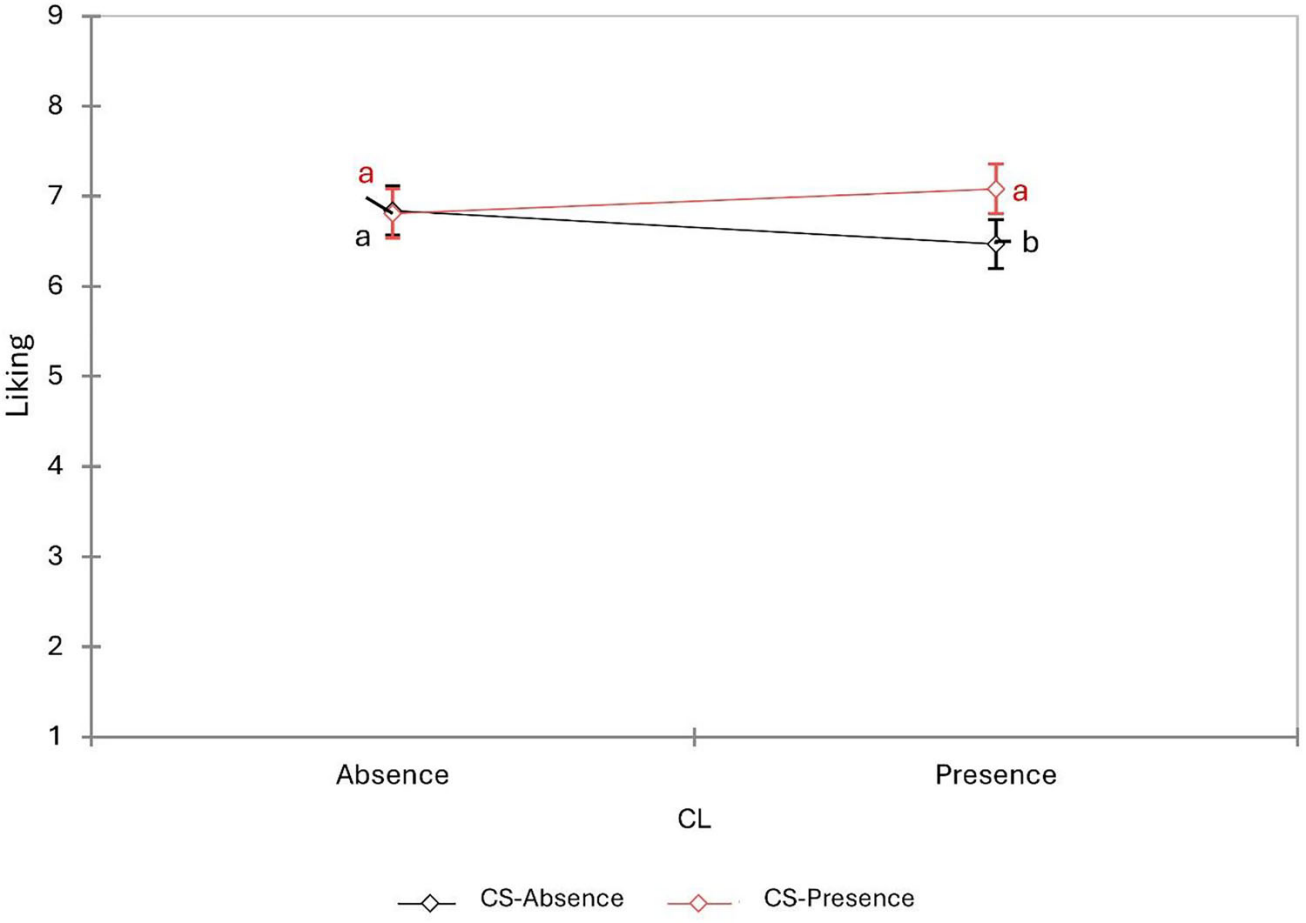
Interaction plot of average liking scores for presence or absence of CL and CS in chocolates in stimulated conditions. Different letters indicate statistical differences between absence and presence of CS for each CL condition.

When consumers were exposed to FOPL designed based on the hypnotized samples in Table 1 (Figure S1), without tasting the chocolate samples again, the two‐way ANOVA results showed that CS (*p* = 0.035) and CS × CL (*p* = 0.021) significantly affected the expected liking of the chocolates and CL did not (*p* = 0.729). Figure 3 shows that when FOPLs were without CL elements (Figure S1A,B), the presence of CS did not significantly impact the expected liking. On the other hand, when CL elements were present on the chocolate FOPL, the indication of “with fibers from cocoa beans” significantly increased the liking scores.

CL foods have been tracked to higher consumer preferences mainly because of the perception of quality and nutritional aspects (Almeida Sá et al. 2023). In addition, consumers tend to avoid foods with unfamiliar ingredients and synthetic additives, preferring foods with simple/short ingredient lists, which leads consumers to infer the cleanness and naturalness of food products (Asioli et al. 2017). However, the present results show that solely clean label did not influence consumers’ acceptance, although it significantly influenced product acceptance when it was tasted (Figure 1B). In the context of upcycled foods, the results of the present study showed that the presence of an upcycled ingredient element on FOPL significantly impacts consumers’ attitudes, since consumers present higher liking when they are exposed to the chocolate package, but the taste of the product is not impacted (Figure 1A). The use of food residues is perceived as a sustainable and nutritional approach for human consumption (Maschio et al. 2023). Nguyen et al. (2025) observed that informing consumers about the nutritional and environmental benefits of upcycled biscuits added to brewery by‐products enhances purchase intent, but may compromise repurchase intent, with the effect driven by sensory perception. In the present work, however, if CL elements were not present on chocolate FOPL, the indication of CS on FOPL did not influence expected liking; however, if CL was present, the addition of CS fibers increased the expected liking scores (Figure 3). To the best of our knowledge, this is the first report on the interaction between clean labels and upcycled cues on food acceptance. The results herein indicate a synergy of this cue with naturalness and less processed perception that CL brings to consumers’ minds, and further studies should explore this phenomenon in depth.

It is important to note that, after consumers taste chocolate samples, CL negatively impacts sensory acceptance (Figure 1B), and food industries should consider consumers’ expectation theories. The contrast theory states that the consumer maximizes the disparity between the tasted product and what he expected initially, and when the expectation is not met by the current performance of the product, the consumer will have a less favorable evaluation in comparison to no prior expectations (Cardello and Sawyer 1992). Thus, since consumers presented higher liking for the indication of CS in the chocolate packaging, but the upcycled ingredient did not influence the acceptance (Figure 1A), food industries must be caution to possible contrast effect when launching these kinds of products. Similar concern must be given for the combination of CL and CS, since the presence of CL elements did not influence expectation (Figure 3), but CL reduced liking (Figure 1B). However, further work must be performed to better explore and understand consumers’ attitudes toward these contrasting results.

#### 3.3.2 Emotions

The effect of the chocolates’ FOPL with different stimuli on the emotions evoked is shown in Table 3. In contrast to the results observed in the blind tests, when consumers were stimulated by FOPL, the presence/absence of CS elements had the most dominant effect on the emotions evoked. “Understanding” (*p* < 0.001), “Enthusiastic” (*p* = 0.000), “Happy” (*p* = 0.041), “Interested” (*p* < 0.001), “Satisfied” (*p* = 0.017), “Secure” (*p* = 0.021), and “Tame” (*p* < 0.001) were affected by CS elements. In contrast, “Good‐natured” (*p* = 0.026) and “Secure” (*p* = 0.021) were affected by CL elements on the FOPL. “Aggressive” (*p* = 0.018) was affected by the interaction of CS × CL.

**TABLE 3:** Deviance and associated *p*‐values from GLM associated with effect of cocoa shell (CS), clean label (CL), and their interaction by emotion for chocolate samples FOPL stimuli. Bold values indicate significance at 5% of significance.

Emotions	CS		CL		CS × CL	
	Deviance	*p*‐value	Deviance	*p*‐value	Deviance	*p*‐value
Affectionate	0.292	0.589	0.571	0.449	2.733	0.098
Loving	0.661	0.416	1.091	0.296	0.081	0.777
Active	0.210	0.647	0.472	0.492	0.442	0.506
Adventurous	3.064	0.080	1.460	0.227	0.131	0.717
Good	0.904	0.342	0.067	0.795	0.185	0.667
Good‐natured	3.125	0.077	**4.980**	**0.026**	0.001	0.970
Calm	0.213	0.644	1.438	0.230	0.662	0.416
Understanding	**12.700**	**< 0.001**	0.931	0.334	1.488	0.223
Enthusiastic	**11.388**	**0.000**	0.799	0.372	1.374	0.241
Happy	**4.164**	**0.041**	1.764	0.184	0.853	0.356
Interested	**23.356**	**< 0.001**	2.753	0.097	3.097	0.078
Mild	1.644	0.200	0.033	0.855	0.036	0.849
Free	0.396	0.529	0.703	0.402	0.032	0.858
Nostalgic	7.497	0.006	4.846	0.028	1.369	0.242
Satisfied	**5.717**	**0.017**	1.320	0.251	0.854	0.356
Secure	**5.351**	**0.021**	**5.298**	**0.021**	1.102	0.294
Aggressive	0.000	1.000	0.000	1.000	**5.576**	**0.018**
Disgusted^a^	**—**	**—**	**—**	—	—	—
Tame	**32.214**	**<0.001**	3.701	0.054	0.000	0.983
Guilty	0.765	0.382	1.727	0.189	1.389	0.239
Worried	0.133	0.715	0.000	1.000	0.133	0.715

^a^ Disgusted was analyzed from analysis since it presented zero citation.

**FIGURE 4:**
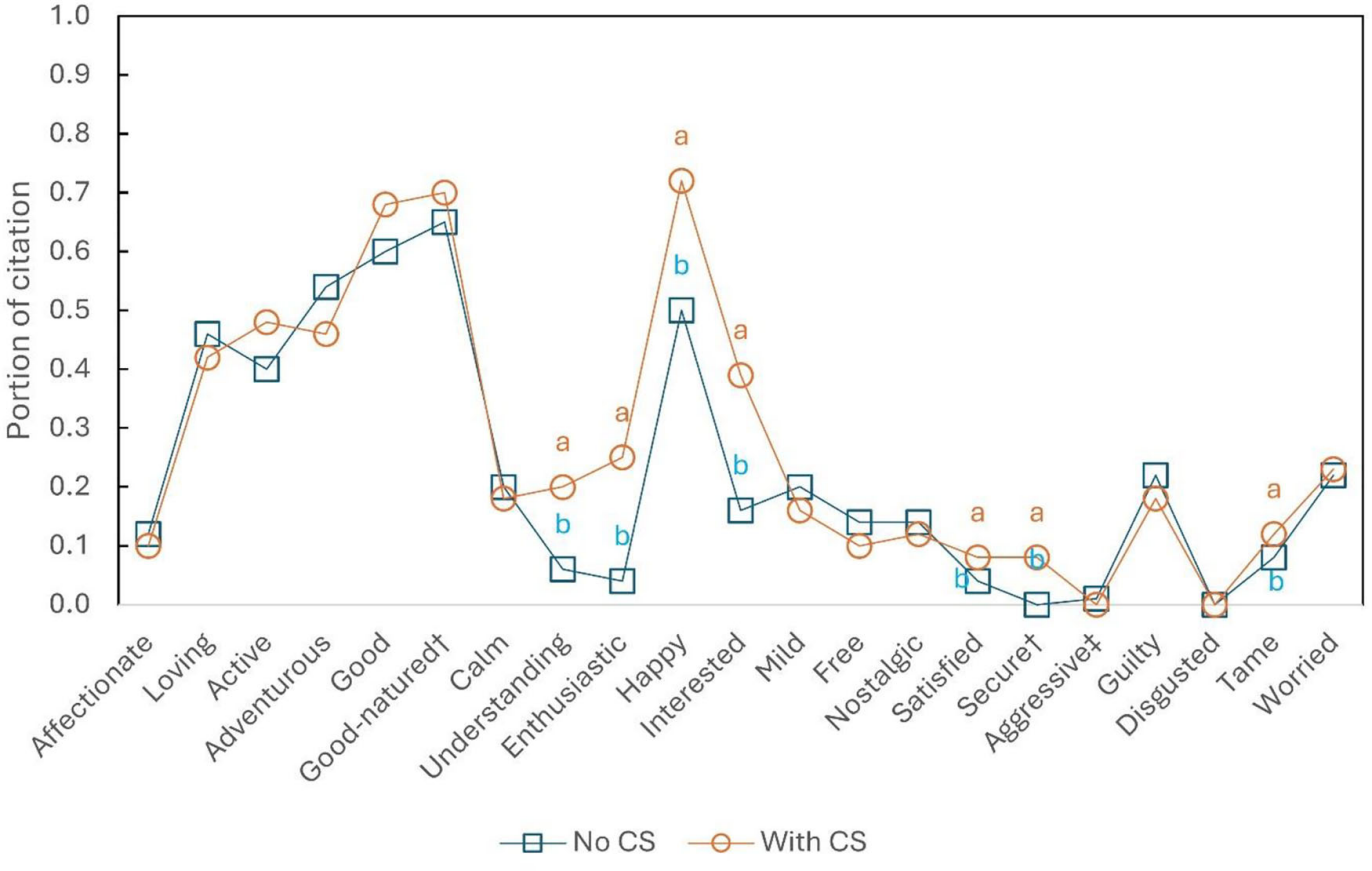
Portion of citation of emotions significantly different between the presence (orange line) and absence (blue line) of CS elements in front‐of‐package labels. Different lowercase letters are significantly different across presence/absence of CS at 5% level according to Tukey's HSD test. When letters are not shown, it means not significant. † indicates significant differences between CL elements within that emotion term. ^‡^ indicates significant interaction (CS × CL) identified in the analysis of deviance.

For all emotions with significant differences between the presence or absence of CS elements on FOPL, a higher frequency of citations was observed when CS elements were present (Figure 4). “Enthusiastic,” “Happy,” “Interested,” “Satisfied,” and “Secure” are positive emotions (King and Meiselman 2010) and results indicate that the indication on chocolates’ FOPL evoked positive emotions. “Understanding” and “Tame” depends on food and context (King and Meiselman 2010), and results herein indicate that they present positive connotation. “Secure” was also significant in the presence/absence of CL (*p* = 0.021) and higher portion of citation was observed when CL elements were present on FOPL. The post hoc Tukey's test to evaluate the influence of CS × CL toward “Aggressive” showed no statistical differences, possibly due to low frequency of citation of the emotion term like observed earlier (Dupas de Matos et al. 2025).

Cattaneo et al. (2018) observed positive attitudes toward breads, tomatoes, apple puree, and yoghurt with grape by‐product extract. The authors also observed that informing consumers about the benefits and concerns regarding food by‐products can result in a more positive attitude toward the use of food residues. De Barcellos et al. (2012, 2015) indicated that consumers have strong collectivist values ​​and very positive attitudes toward the environment, which positively influences the purchase of eco‐innovative foods. Thus, the communication that accompanies by‐products as ingredients and waste‐based products among consumers can result in better knowledge and, consequently, reach consumers and resonate with sustainable thoughts.

The differences observed between blind and informed conditions suggest that consumers’ emotional responses were not driven exclusively by sensory perception, but cognitive expectations may also strongly modulate food choices generated by the FOPL information. In blind tests, emotions such as “Affectionate” were significant cited because consumers based their evaluations primarily on the multisensory eating experience, including flavor, texture, and overall hedonic pleasure. Emotional terms with interpersonal or comfort‐related meanings, such as “Affectionate” and “Loving,” are commonly associated with indulgent and pleasurable chocolate consumption experiences (King and Meiselman 2010; Nestrud et al. 2016). However, when FOPL stimuli were presented, consumers’ attention may have shifted from purely hedonic perception toward more cognitive and functional product interpretations, particularly regarding sustainability, naturalness, healthiness, and ingredient awareness associated with cocoa shell and clean label claims. Consequently, emotions linked to cognitive appraisal, such as “Understanding,” “Interested,” and “Secure,” became more prominent in the informed condition. The additional information likely changed the consumption context, redirecting consumers’ emotional processing from emotional comfort and pleasure toward rational evaluation and product interpretation. Simultaneously, the increase in positive emotions such as “Interested,” “Enthusiastic,” and “Secure” suggests that the presence of cocoa shell and clean label cues generated curiosity, engagement, and perceptions of transparency or trustworthiness. These findings reinforce that packaging information can reshape emotional responses by activating consumers’ prior beliefs, expectations, and values before tasting, highlighting the important role of FOPL as a modulator of emotional perception in chocolate products.

#### 3.3.3 Laddering

In the hard laddering task, consumers were initially asked to indicate which chocolate they would purchase based on the FOPL (Figure S1). Whole statistical description is presented in Supporting Information. The base sample was chosen by 5.9% (*n* = 8), CS by 27.4% (*n* = 37), CL by 13.3% (*n* = 18), and CS + CL by 53.3% (*n* = 72). The combination of CS and additives with a clean label appears to synergistically promote a more positive perception, directly influencing the choice of the product. These findings corroborate previous studies that highlight the positive impact of clear information and attributes perceived as healthy on purchase decisions for processed foods (Almeida Sá et al. 2023). The use of interpretive messages and the combination of functional and sustainable attributes can generate stronger emotional appeal and higher purchase intention, as reflected in the consumer responses in this study. CS + CL was the most chosen chocolate and high acceptance of FOPL (Figure 3), but the lack of chemicals in the chocolate formulation (CL chocolate) implies lower acceptance (Figure 1B). Thus, food industries must pay attention in these scenarios to minimize expectation contrast effects. The laddering results also help explain the emotional differences observed between blind and stimulated conditions. During blind evaluation, consumers relied primarily on the sensory experience, which was associated with hedonic and affective emotions such as “Affectionate,” “Loving,” “Happy,” and “Satisfied.” In contrast, when consumers were exposed to FOPL information, the laddering task revealed that product choice was strongly driven by “more information,” “knowledge,” “trust,” “health,” and “sustainability.” These drivers are closely aligned with the higher citation frequencies of cognitive and value‐oriented emotions such as “Interested,” “Enthusiastic,” “Understanding,” and “Secure” observed in the stimulated tests. Therefore, the laddering findings provide a behavioral explanation for the emotional shift from predominantly hedonic responses in blind tasting to more cognitive and value‐based responses when packaging information was available.

**FIGURE 5:**
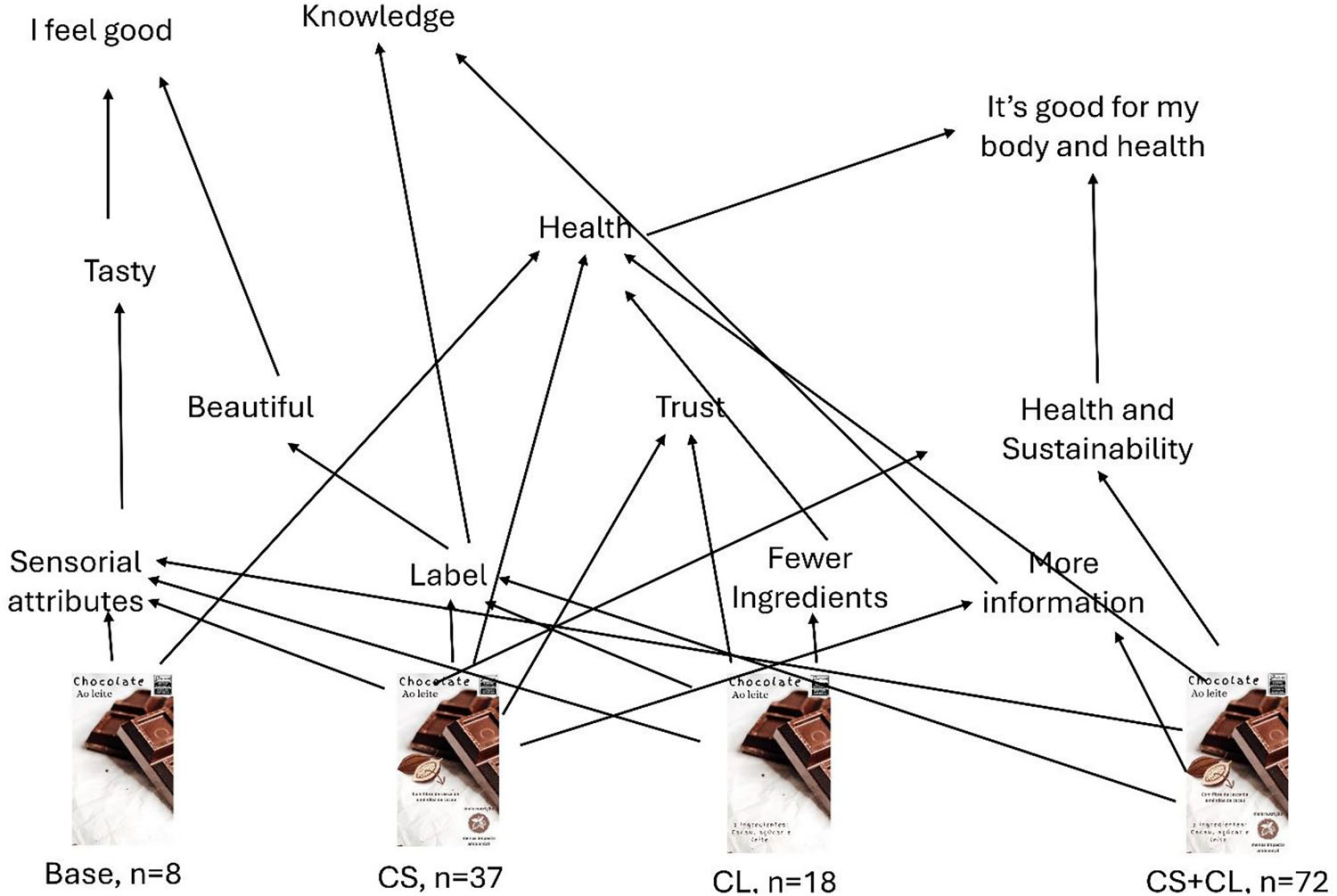
Hierarchical value map based on consumers’ choice.

Figure 5 shows a hierarchical value map based on consumer choices. Preference for the control sample was highly linked to sensory attributes, which were weaker in the other FOPL samples than in the control. The CS FOPL was highly linked to healthy aspects and both health and sustainability aspects, which were also linked to the CS + CL stimulus. The choice of CL FOPL was equally related to fewer ingredients, labels, trust, and sensorial aspects. CS + CL was highly related to more information presented on FOPL, although its choice was also linked to fewer ingredients, trust, health, and label issues. More information was related to knowledge, which indicates consumers’ willingness to know what they were eating. Sensorial aspects were tracked to hedonic issues (taste) and make consumers feel good. Health and sustainability were linked to making good for the body and health of consumers, while more information was related to consumers’ knowledge of what they would be eating.

Almeida Sá et al. (2023) related consumers’ purchasing intention of CL‐processed foods with perception of quality and nutritional values, and observed that consumers paid attention to the list of ingredients disposed on products’ FOPL. In addition, Brazilian consumers trust the information provided on FOPL (De Sousa et al. 2020), which helps to explain tracking CS and CS + CL to more information as a driver of purchasing. Regarding health, there is no standard definition of CL, but studies have shown that consumers relate it to natural ingredients (Aschemann‐Witzel et al. 2019), and naturalness is usually linked to health‐promoting aspects (Rivaroli et al. 2020). As described by Barrena et al. (2015), health aspect (linked to CS, CL, and CS + CL) is a psychological consequence of food choice that enhances quality of life and security consumers’ perception, which is in line with the findings of the present study. Sustainable perception of utilization of food by‐products into food formulation has also been reported previously by Maschio et al. (2023), who found that cakes added with banana peel are perceived as more sustainable than the conventional bakery. Thus, the results indicate that food industries must design FOPL with more and clearer nutritional information, looking at natural ingredients, and value food by‐products if they are added to the formulation to enhance consumers’ willingness to purchase and evoke positive feelings and perceptions. Further works should explore the FOPL and these drivers of purchasing as claims onto the label to evaluate their effect on expectations, liking, acceptance, and preferences.

## 4 Conclusion

In conclusion, CS and CL affected the acceptance, expected liking, and/or purchasing intentions of chocolates. The absence of additives may negatively impact hedonic aspects, while the addition of cocoa fibers has no influence on acceptance, and positive emotions evoked during consumption lead to higher acceptance. However, the disposal of cocoa fibers onto FOPL positively impacted acceptance and evoked more positive emotions, and the presence of CL elements did not impact expected liking. These contrasts in expected and real liking must be paid attention by food industries to avoid contrast effects on consumers’ attitudes and behaviors. The results showed that the combination of CS and CL on FOPL was the most preferred chocolate, and the presence of more information regarding food composition was the main driver of choice, since consumers want to have knowledge of what they are consuming.

The findings of this study provide important insights for future trends in Food Science and Technology, particularly regarding the development of sustainable, consumer‐oriented, and emotionally engaging food products. The demonstrated synergy between clean label strategies and upcycled cocoa shell ingredients highlights a promising pathway for the design of next‐generation chocolates that align simultaneously with sustainability, transparency, and health‐related consumer expectations. Moreover, the results reinforce the growing importance of front‐of‐pack communication and emotional responses as key drivers of food choice, extending beyond traditional sensory acceptance alone. As consumers increasingly demand environmentally responsible products with natural and understandable ingredients, the integration of upcycled by‐products combined with effective labeling strategies may become a major innovation trend in the food industry. These findings also suggest that future research should further explore the interaction between sensory perception, emotional profiling, sustainability cues, and consumer expectations to support the development of more competitive and sustainable food systems.

As limitations, consumer tests were carried out in a controlled environment and did not represent reality, impacting the results presented. In addition, fictitious chocolate labels were used to stimulate consumers toward CS and CL, which could impact their overall perception. Although the present study identified specific emotions significantly affected by chocolate formulation and front‐of‐package information, the statistical relationships among emotions themselves were not investigated. Future studies could apply multivariate approaches to explore co‐occurrence patterns and latent emotional dimensions. Such approaches may provide a deeper understanding of how emotional responses are organized and how different emotional constructs jointly influence consumer acceptance, purchase intention, and food choice.

However, the present work provides innovative information of great relevance to the food industry, and limitations may not negate the intrinsic value of this work.

## Author Contributions

**Fernanda Schiehll Azevedo**: conceptualization, investigation, writing – original draft, visualization. **Voltaire Sant'Anna**: conceptualization, writing – review and editing, validation, methodology, formal analysis. **Juliana Santos**: conceptualization, supervision, project administration, writing – original draft. **Ludmylla Tamara Crepalde**: writing – review and editing, software, formal analysis, data curation. **Marc François Richter**: conceptualization, writing – review and editing, supervision, project administration.

## Conflicts of Interest

The authors declare no conflicts of interest.

## Supporting information




**Supplementary Information**: jfds71360‐sup‐0001‐SuppMat.docx

## Data Availability

The experimental data obtained in the current study are available from the corresponding author on reasonable request.
