# Supplementary material for: Exploring Consumers’ Acceptance, Emotions, and Attitudes Toward Clean Label Chocolates and/or Added Cocoa Shell
Source: J Food Sci. 2026 Aug 17;91(8):e71360. doi: 10.1111/1750-3841.71360 (PMC13481851; doi:10.1111/1750-3841.71360)
Supplement: Supplementary file 1 [file JFDS-91-0-s001.docx]

**Supplementary material**

**Table S1.** Factorial design with code variables (real variables) to produce chocolate samples for consumers’ tests.

| Sample | CS | CL |
| --- | --- | --- |
| BS | -1 (absence – without CS) | -1 (No - with additives) |
| CS | +1 (presence – with CS) | -1 (No - with additives) |
| CL | -1 (absence – without CS) | +1 (Yes - without additives) |
| CS+CL | +1 (presence – with CS) | +1 (Yes - without additives) |

BS: base sample; CS: cocoa shell; CL: clean label

**Table S2.** Consumers’ profile involved in sensory analysis (N=135)

|  | n | f (%) |
| --- | --- | --- |
| **Gender** |  |  |
| Male | 52 | 38.5% |
| Female | 83 | 61.5% |
| **Education level** |  |  |
| Incomplete fundamental school | 1 | 0.7% |
| Complete fundamental school | 5 | 3.7% |
| Complete high school | 30 | 22.2% |
| Complete College | 41 | 30.4% |
| Post-graduation | 57 | 42.2% |
| Did not respond | 1 | 0.7% |
| **Age** |  |  |
| Mean | 38.2±10.1 years old | |
| Maximum | 65 years old | |
| Minimum | 18 years old | |
| **Monthly income^†‡^** |  |  |
| Less than 1 minimum salary | 3 | 2.2% |
| Between 1 and 3 minimum salaries | 45 | 33.3% |
| Between 3 and 5 minimum salaries | 31 | 23.0% |
| Between 5 and 7 minimum salaries | 22 | 16.3% |
| More than 7 minimum salaries | 33 | 24.4% |
| Did not respond | 1 | 0.7% |
| **Frequency of consumption** |  |  |
| Everyday or almost everyday | 32 | 23.7% |
| Several times a week, but not every day | 28 | 20.7% |
| Once a week | 32 | 23.7% |
| Several times a month but not every week | 27 | 20.0% |
| Once a month | 4 | 3.0% |
| Several times a year, but not every month | 10 | 7.4% |
| Once or twice a year | 2 | 1.5% |

^†^U$ 1.00 ~R$ 5.84, in February 2nd 2025.

^‡^ minimum salary in Brazil in 2024 was R$1.412,00 monthly

**Figure S1.** Chocolates’ front-of-packages used as stimuli to evaluate expected liking and emotions evoked by control chocolates (A), chocolates added of cocoa shell (B), clean label chocolate (C) and clean label chocolate added of cocoa shell (D).

**Suplementary material 1.** Statistical analysis of chocolale FOPL from section 3.2.3

Consumers’ choices among the four chocolate samples differed significantly, as evidenced by the chi-squared test (χ² =76.66; *p*<0.001). The analysis of the adjusted standardized residuals allowed us to identify which samples contributed significantly to this result, where the CS + CL sample presented a highly positive residual value (z = 6.77), being significantly more chosen than expected. In contrast, the control sample (C) was the least chosen, with a significant negative residual (z = –4.69), followed by the CL sample (z = –2.87), which was also significantly below expected. The CS sample did not present a significant deviation (z = 0.79), indicating that the frequency of choice was close to the expected value under the null hypothesis. In general, values above 1.96 (or below -1.96) indicate statistically significant differences (5% level) (MacDonald and Gardner 2000; Sharpe 2015; Kim 2017). When performing multiple comparisons between proportions, it was observed that the CS + CL sample was significantly more preferred than all the others (*p*<0.001), while sample C showed a significant difference in relation to CS (*p*=0.000003), CL (*p*=0.26), and CS + CL (*p*<0.001).
